# A Potential Atypical Case of Rabbit Haemorrhagic Disease in a Dwarf Rabbit

**DOI:** 10.3390/ani11010040

**Published:** 2020-12-28

**Authors:** Fábio A. Abade dos Santos, Carolina Magro, Carina L. Carvalho, Pedro Ruivo, Margarida D. Duarte, Maria C. Peleteiro

**Affiliations:** 1 Centre for Interdisciplinary Research in Animal Health (CIISA), Faculdade de Medicina Veterinária, Universidade de Lisboa, Avenida da Universidade Técnica, 1300-477 Lisboa, Portugal; margarida.duarte@iniav.pt (M.D.D.); mcpelet@fmv.ulisboa.pt (M.C.P.); 2 Instituto Nacional de Investigação Agrária e Veterinária (INIAV, I.P.), Av. da República, Quinta do Marquês, 2780-157 Oeiras, Portugal; carina.carvalho@iniav.pt; 3 Instituto Universitario de Biotecnología de Asturias (IUBA), Departamento de Bioquímica y Biología Molecular, Universidad de Oviedo, 33006 Oviedo, Spain; 4 VetOeiras, Hospital Médico-Veterinário, Estrada de Oeiras n18-20, 2780-114 Oeiras, Portugal; carolinamagrovet@gmail.com; 5 Instituto de Medicina Molecular João Lobo Antunes (IMM), Faculdade de Medicina, Universidade de Lisboa, 1070-312 Lisbon, Portugal; ruivo_pedro@hotmail.com

**Keywords:** European rabbit, *Oryctolagus cuniculus*, pet rabbit, rabbit haemorrhagic disease, atypical clinical course, subacute

## Abstract

**Simple Summary:**

We report an unusual clinical case in a pet rabbit vaccinated against rabbit haemorrhagic disease virus (RHDV, GI.1), that developed a prolonged hepatic disease, and was diagnosed RHDV2 (GI.2) positive post-mortem. This finding is a warning to all veterinarians that rabbit haemorrhagic disease should also be considered for differential diagnosis despite the history of RHDV vaccination and the need to update vaccination programs against the current RHDV2 circulating strains.

**Abstract:**

Rabbit haemorrhagic disease (RHD) is a highly contagious infectious disease of European wild and domestic rabbits. Rabbit haemorrhagic disease virus (RHDV, GI.1) emerged in 1986 in Europe, rapidly spreading all over the world. Several genotypes of RHDV have been recognised over time, but in 2010, a new virus (RHDV2/RHDVb, GI.2) emerged and progressively replaced the previous RHDV strains, due to the lack of cross-immunity conferred between RHDV and RHDV2. RHDV2 has a high mutation rate, similarly to the other calivirus and recombines with strains of RHDV and non-pathogenic calicivirus (GI.4), ensuring the continuous emergence of new field strains. Although this poses a threat to the already endangered European rabbit species, the available vaccines against RHDV2 and the compliance of biosafety measures seem to be controlling the infection in the rabbit industry Pet rabbits, especially when kept indoor, are considered at lower risk of infections, although RHDV2 and myxoma virus (MYXV) constitute a permanent threat due to transmission via insects. Vaccination against these viruses is therefore recommended every 6 months (myxomatosis) or annually (rabbit haemorrhagic disease). The combined immunization for myxomatosis and RHDV through a commercially available bivalent vaccine with RHDV antigen has been extensively used (Nobivac^®^ Myxo-RHD, MSD, Kenilworth, NJ, USA). This vaccine however does not confer proper protection against the RHDV2, thus the need for a rabbit clinical vaccination protocol update. Here we report a clinical case of hepatitis and alteration of coagulation in a pet rabbit that had been vaccinated with the commercially available bivalent vaccine against RHDV and tested positive to RHDV2 after death. The animal developed a prolonged and atypical disease, compatible with RHD. The virus was identified to be an RHDV2 recombinant strain, with the structural backbone of RHDV2 (GI.2) and the non-structural genes of non-pathogenic-A1 strains (RCV-A1, GI.4). Although confirmation of the etiological agent was only made after death, the clinical signs and analytic data were very suggestive of RHD.

## 1. Introduction

Originating in the Iberian Peninsula [1], the European rabbit was widely introduced first in Europe, and subsequently in all other continents except Antarctica. The European domestic rabbit has been used as an important meat and fur resource. Artificial selection of small breeds of this species (*Oryctolagus cuniculus*), weighing less than 2 kg, also generated an increasingly popular pet-rabbit trade. Pet rabbits can be affected by the same diseases found in the rabbit industry or wild rabbit populations. However, the indoor lifestyle decreases the risk of contact with common rabbit diseases such as myxomatosis and rabbit haemorrhagic disease (RHD).

RHD is a highly contagious infectious disease caused by a virus from the Caliciviridae family, genus *Lagovirus*, which targets hepatocytes and cells of the mononuclear phagocytic system (e.g., Kuppfer cells and alveolar macrophages) [2,3]. The disease was first identified in 1984 in China and quickly spread to other continents, evolving into different genotypes (GI.1a (RHDVa or G6), GI.1b (G1), GI.c (G2) and GI.1d (G3–G5)) [4,5]. Along with myxomatosis, this disease led to an abrupt decrease in the European rabbit wild populations [6].

In 2010, a distinct virus (both genetic and antigenically) related to RHDV emerged in France [7]. This new virus, referred to as RHDV2 or RHDVb, induced a disease very similar to that caused by RHDV strains and it became also known as *Lagovirus europaeus* GI.2 [5].

**Figure 1:**
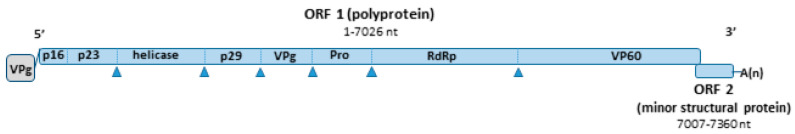
Simplified schematic representation of the genomic RNA of RHDV2. Nucleotide positions were calculated from sequence KF442964.2. The genome includes two ORFs. ORF1 encodes a polyprotein that is cleaved to form the non-structural proteins p16, p23, the helicase, p29, VPg, a protease and the viral polymerase, and the major structural protein VP60. ORF 2 encodes a minor structural protein VP10.

More recently, natural recombinant RHDV2 strains were identified containing the structural proteins of RHDV2 (VP60 and minor protein-encoding genes), but the non-structural proteins from either non-pathogenic calicivirus (NP-CV GI.4 and GI.3)). Furthermore, the non-structural proteins from the RHDV strains (GI.1b) were also found in RHDV2 recombinants [8]. Figure 1 represents the genomic organization of RHDV2 RNA.

Different clinical presentations and disease progression can occur with RHDV2 [9,10,11,12] and those variations can be associated with strain. The incubation period of RHD ranges between 1 and 3 days. In peracute forms, sudden death may occur within 12 hours after infection. In acute and subacute forms of RHD, different clinical signs can be observed, such as anorexia, mucosal congestion, neurologic signs, cyanosis, dyspnoea, foamy haemorrhagic epistaxis, ocular haemorrhage and others [13]. In chronically infected rabbits, the onset of anorexia, lethargy and jaundice usually precedes death in about 1 to 3 weeks [13]. Chronic or subclinical courses are more frequent in rabbits infected by RHDV2 than by RHDV.

Haemorrhagic diathesis is found during the disease, with disseminated intravascular coagulation (DIC), associated with alterations in blood coagulation and terminating in multi-organ failure and death [14]. The pathogenesis of DIC in RHD is still unknown. The DIC is assumed to occur by a distortion of both external and internal blood coagulation activation paths, leading to extended One-Stage Prothrombin Time (OSPT) and Activated Partial Thromboplastin Time (APTT) [15,16,17]. During the disease, a thrombocytopenia and reduced platelet aggregation is observed, alteration of the activity of coagulation factors V, VII and X, as well as increased volume of soluble fibrin and its degradation products (D-dimers) [15,16,17].

During an RHDV2 outbreak, more than 10% of the infected rabbits may show a chronic or subclinical evolution of the disease, with severe and generalised jaundice, loss of weight, and lethargy. These animals often die some weeks later, due to liver disease [18]. Although the study of the disease has been expanded by animal experimentation, the characterisation of natural infection is far less known [10,11,19,20].

The terms peracute, acute and chronic are of clinical use but not always reflected in distinct necropsy findings and specific histopathological patterns, meaning that it is possible to find sudden death associated with peracute widespread hepatocellular necrosis or with more chronic changes such as inflammation or fibroplasia [10].

Veterinarians should be aware that despite necropsy and histopathology findings may be suggestive of RHD, since microscopic changes are usually present and, in many cases, macroscopic changes can also be seen [10], laboratory confirmation is always required for a conclusive diagnosis.

Laboratory testing can be done by molecular methods (e.g., RT-PCR), ELISA or electron microscopy. However, the rapid and debilitating evolution of the disease, together with the shortage of direct access to these techniques by clinicians, poor reporting of RHD in pet rabbits and possible lack of awareness among clinicians, contributes to the almost non-existent knowledge of the prevalence of RHD in pets in Portugal.

After the emergence of RHDV2 in 2010, this virus rapidly replaced the previous strains that soon were no longer reported in Europe. The humoral immunity acquired by natural- or vaccine-induced RHDV strains do not confer proper cross-protection against RHDV2 strains [21,22]. Until 2016, when ERAVAC, Hipra, Girona, Spain was commercialised, all vaccines available against RHDV2 were directed to the industrial market (sold in multi-dose bottles), and therefore not adequate for vaccination of pet rabbits. In November 2019, a new single-dose bottle vaccine against myxoma virus, RHDV and RHDV2 was also made available in Europe (Nobivac^®^ Myxo-RHD Plus, MSD, Kenilworth, NJ, USA).

## 2. Clinical Presentation

We present the case of a 2-year-old spayed female Netherland dwarf rabbit. This rabbit was dewormed yearly with fenbendazole, (Panacur®, Merck Animal Health, Giralda Farms, Madison, WI, USA, 20 mg/kg) and twice a year with a 15 mg selamectin spot-on (Stronghold®, Zoetis, Belgium vaccinated yearly (Nobivac® Myxo-RHD, MSD, Kenilworth, NJ, USA), and was kept indoors with an owner residing in the Oeiras District, Portugal. At the age of 18 months, the patient was presented for annual vaccination. Physical examination at that point was unremarkable and the rabbit was clinically normal.

In February 2020, 2 months after the previous visit, the rabbit presented for anorexia, coprostasis and lethargy. The physical examination only revealed a painful condition as the rabbit exhibited bruxism (teeth grinding) after cranial abdominal palpation. The rest of the physical examination was unremarkable and the rabbit weighed 1580 g at the time. A complete blood count (CBC, detailed in Table 1) revealed leukopenia (2.53 K/µL) with heteropenia (0.71 K/µL) and thrombocytopenia (40 K/µL). Alanine aminotransferase (ALT) levels were elevated (240 U/L), indicating liver damage and radiographs showed a more radio-opaque cranial abdomen. The patient was admitted and started on standard intravenous fluid therapy with NaCl 0.9%, was administered buprenorphine (0.03 mg/kg IV TID) for analgesia and provided nutritional support (Oxbow’s herbivore critical care), recovering appetite about 6 hours later.

Ultrasound revealed a slightly heterogeneous liver with a hypoechoic caudate lobe and a kidney stone considered meaningless for this case. There were no obvious signs of vascular compromise. However, shaving the abdomen for the ultrasound exam revealed a large haematoma that extended from the neck to forelimbs, thorax and upper-half of the abdomen. This was assumed to have originated from the jugular blood collection carried out in the previous day, despite the seemingly atraumatic puncture.

Throughout the 5th day of hospitalisation, the patient maintained a pattern of cutaneous haemorrhagic dyscrasia, with vascular fragility, easy bruising and requiring the need for prolonged compression of puncture sites. Coagulation tests were carried out and both prothrombin time (PT) and activated partial thromboplastin time (aPTT) were prolonged, at 25 and 195 s, respectively.

A CT scan showed no signs of hepatic abnormalities or vascular compromise. The patient was started on injectable vitamin K1 (10 mg/kg SC SID) and kept on buprenorphine (0.03 mg/kg SC TID) and IV NaCl 0.9% at a rate of 5mL/h. Bruising was fully reabsorbed by the 8th day and the patient was sent home. Before release, the physical exam was unremarkable. The rabbit was bright and alert and had fully recovered his appetite, weighting at the time 1500 g. No more blood collections were possible due to lack of venous accesses, secondary to multiple hematomas and bruising in the previous blood collection sites. Two weeks later, in March 2020, follow up blood tests were performed, including a CBC. The revaluation hemogram was unremarkable, as reticulocytosis was considered a normal finding given the events in the previous hospitalisation. No other clinical follow up could be performed due to financial limitations and lockdown policies associated with the COVID-19 world pandemic.

**Figure 2:**
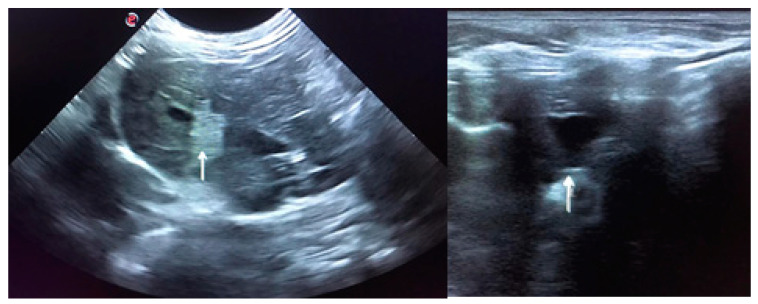
Ultrasound is compatible with hepatitis. The image on the left shows peri-lobular mesenteric reactivity (arrow). On the right, free fluid in the abdomen is visible (arrow).

In June 2020, the rabbit was readmitted due to anorexia, lethargy, coprostasis and abdominal discomfort. The rabbit had lost 80 g, weighting then 1420 g, and for the first time, mildly hyperthermia (39.4 °C) was registered. The blood panel revealed severe leukopenia (1.86 K/μL) associated with heteropenia (0.89 K/μL) and lymphopenia (0.78 K/μL). Thrombocytopenia (97 K/μL) was also observed. ALT was then 586 U/L and there was also the elevation of total bilirubin (0.9 mg/dL) and total proteins (7.7 g/dL) due to hyperglobulinemia (3.3 g/dL) (Table 1). The blood draw resulted once again in cutaneous haemorrhagic dyscrasia and therefore the rabbit was started on vitamin K1 (10 mg/kg SC BID), enrofloxacin (5 mg/kg SC SID), buprenorphine (0.03 mg/kg SC TID), metoclopramide (0.5 mg/kg SC BID), lactulose (0.5 mL/kg PO BID) and aggressive fluid therapy. Ultrasound was compatible with severe hepatitis and peri-lobular peritonitis (Figure 2), with mesenteric reactivity and free fluid in the abdomen. The rabbit was in the hospital for 3 days showing no signs of improvement. The temperature kept increasing reaching a peak of 40.2 °C on the second day of hospitalisation, after which it started dropping and the rabbit became progressively hypothermic. On the 3rd day of hospitalization, the temperature dropped to 36.9 °C, despite the active heating efforts. The rabbit became lethargic, jaundiced and developed vertical nystagmus. The death occurred about 6 h after the onset of these symptoms.

Throughout the clinical evolution of this case, several differential diagnoses were considered. These included hepatic lipidosis, hepatic coccidiosis, liver lobe torsion, bacterial, fungal, and parasitic hepatitis, Encephalitozoon cuniculi infection, Tyzzer’s disease and neoplasia. All these possibilities were excluded by the rabbit’s clinical history, semestral coprology results and deworming history, along with the blood works, radiographs, ultrasounds, CT scan and post mortem data.

The necropsy findings reinforced that the inflammatory and/or infectious process was the most likely differential diagnosis for this patient.

## 3. Material and Methods

### 3.1. Necropsy and Histopathology

The necropsy was performed according to routine procedures, and samples were collected for bacteriology (liver, spleen and lung), histopathology (liver, spleen, stomach, small intestine, pancreas and kidney) and virology (liver). All analyses followed routine procedures.

Histopathology fragments were fixed in 10% neutral buffered formalin, routinely paraffin-embedded, sectioned at 4 µm, and stained with Haematoxylin and Eosin (H&E).

### 3.2. Molecular Analysis

For nucleic acid extraction, a fresh sample of liver was homogenized at 10% (*w*/*v*) with phosphate-buffered saline (PBS) and clarified at 3000 g for 5 min. Total nucleic acids were extracted from 200 μL of the clarified supernatants, using the MagAttract 96 cador Pathogen Kit (Qiagen, Hilden, Germany) in a BioSprint 96 nucleic acid extractor (Qiagen, Hilden, Germany), according to the manufacturer’s protocol.

The rabbit was tested for rabbit haemorrhagic disease virus (RHDV) by conventional PCR [24] and rabbit haemorrhagic disease virus 2 (RHDV2) and myxomavirus (MYXV) by real-time PCR [25,26]. Amplifications were carried out in a Bio-Rad CFX96™ Thermal Cycler (Bio-Rad Laboratories Srl, Redmond, WA, USA), using the One-Step RT-PCR kit (Qiagen, Hilden, Germany) for RHDV2, and the NZYTaq II 2x Colourless Master Mix (Nzytech, Lisbon, Portugal) for MYXV.

cDNA was synthesised with the SuperScript™ IV First-Strand Synthesis System (InVitrogen, Carlsbad, CA, USA) according to the manufacturer’s recommendations, using either oligo(dT)12-18 and random hexamers. Amplification of full VP60 gene and partial RdRp gene was achieved using primers, kits and protocols available in appendix Table S1.

The PCR products were visualised in 2% horizontal electrophoresis agarose gel, purified using the NZYGelpure kit (NZYTECH), and directly sequenced using the ABI Prism BigDye Terminator v3.1 Cycle sequencing kit on a 3130 Genetic Analyser (Applied Biosystems, Foster City, CA, USA). Nucleotide sequences were analysed and assembled into consensus sequences using the Seqscape Software v2.7 (Applied Biosystems, Foster City, CA, USA)., The final 2176 nucleotide sequence including the VP60 complete gene (1740 nt-long) and a 436 nt-long region of the RdRp gene, was deposited in GenBank database and given the accession number MT829254.

### 3.3. Phylogenetic Analysis

To further investigate the relation of this strain to other recombinants characterised previously in Portugal [8], a phylogenetic analysis was carried out by Maximum Likelihood (ML) resourcing to the R software (R Development Core Team, 2009) [27]. Three types of multiple sequence alignments (msa) were used for phylogenetic inference, encompassing a 436 nt region within the RdRp gene, the complete 1740 nt vp60 gene sequences, and the 2176 nt long sequence comprising the two regions mentioned.

Multiple sequence alignments (msa) were generated by MUSCLE through the R software (R Development Core Team, 2009). For each alignment, the appropriate substitution model for ML analysis was determined using the function modeltest. For RdRp tree, the Hasengawa–Kishino–Yano (HKY) model [28] an allowance for the incorporation of invariant sites (I) (HKY+I), showed the lower AIC value and was used to infer phylogenetic relationships. For the ML phylogenetic tree based on the full vp60 gene (1740 nt long) the General Time Reversible (GTR) model [29] with a discrete gamma distribution (+G), (GTR+G) showed the lower AIC value was used to infer phylogenetic relationships. For the ML phylogenetic tree based on a 2176 nt long sequence comprising the terminal 436 nt long region within the RdRp gene and the complete vp60 gene the General Time Reversible (GTR) model [29] with a discrete gamma distribution (+G) and/or an allowance for the incorporation of invariant sites (+I) (GTR+G+I) showed the lower AIC value and was used to infer phylogenetic relationships.

## 4. Results

### 4.1. Necropsy and Histopathology

The necropsy revealed liver congestion and marked lobular pattern, presence of a small amount of free peritoneal fluid and congested lungs with haemorrhagic foci. Both kidneys showed areas of surface retraction accounting for about 20% of the total surface.

**Figure 3:**
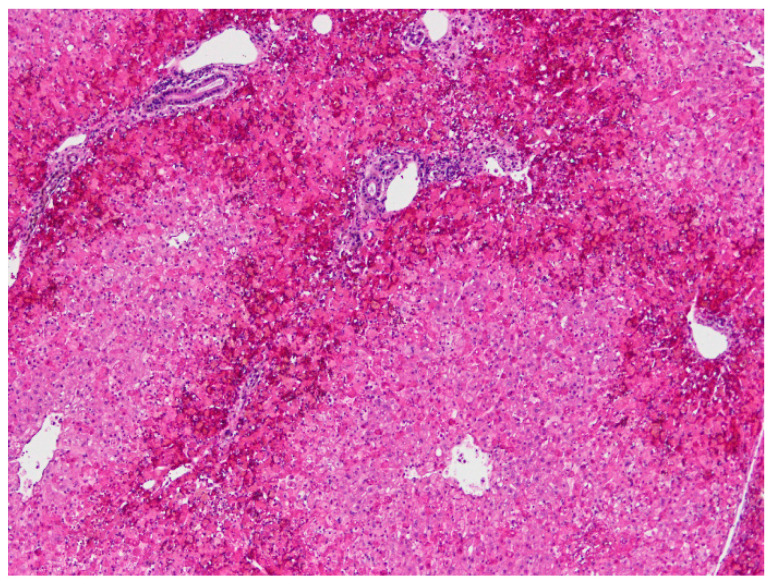
Liver. Severe haemorrhagic necrosis consistently affects perilobular areas or acinar zone 1, conspicuous due to the bright red colour close to the portal areas (H&E, 40×).

**Figure 4:**
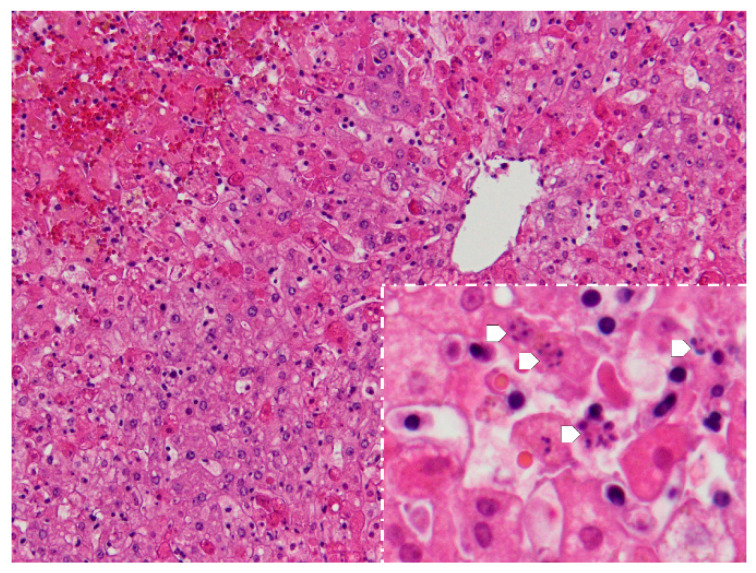
Liver. Apart from perilobular haemorrhagic necrosis, single-cell necrosis is present in dispersed hepatocytes in acinar zones 2 and 3, which surround the periacinar vein in the upper right. Inset-magnification of the acinar zones 2 and 3, showing various cells with the fragmentation of the nucleus-karyorrhexis (arrows) (H&E, 100×, inset 400×).

**Figure 5:**
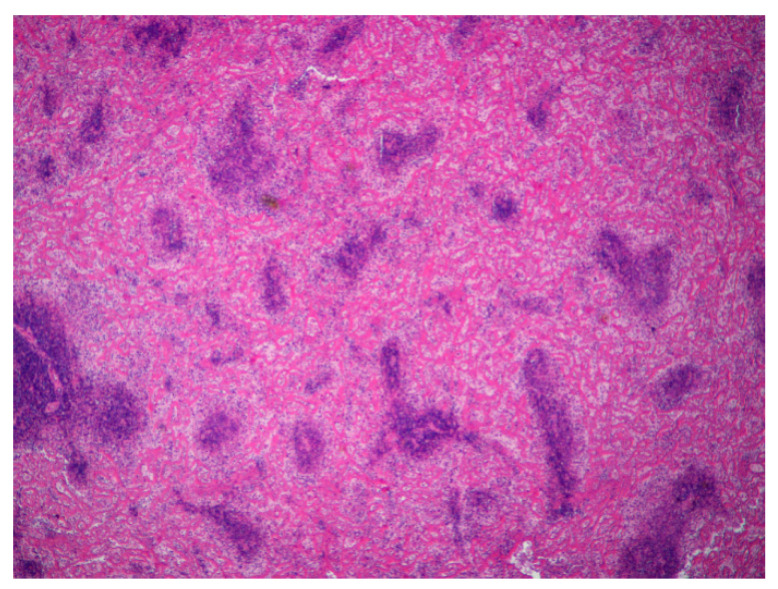
Spleen. Severe diffuse fibrinoid necrosis of the red pulp. The eosinophilic material fibrinoid material is very abundant between the lymphoid tissue that surrounds blood vessels (H&E, 40×).

**Figure 6:**
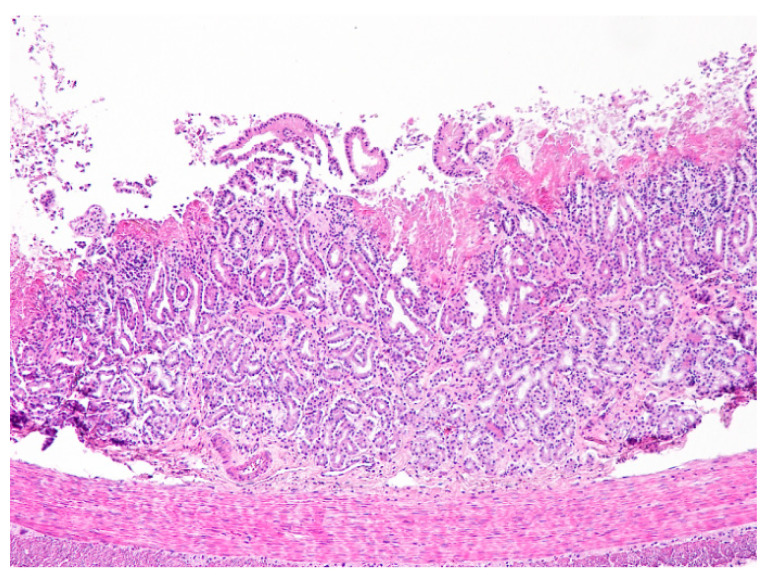
Duodenum. Necrotic enteritis affecting the upper mucosa. Note the absence of villi and the eosinophilic deposits of fibrin in the upper mucosa. The intestinal glands are disorganized and the inflammatory infiltrate of the mucosal lamina propria by mononuclear cells (lymphocytes mostly) is scant (H&E, 100×).

The following microscopic lesions were observed: **Liver**: severe generalized perilobular haemorrhagic necrosis (Figure 3 and Figure 4). Discrete infiltration by mononucleated inflammatory cells, mainly macrophages and lymphocytes, around portal triads. Fine brown pigment in the cells of the portal bile ducts. **Spleen**: diffuse necrosis of the entire red pulp revealed by deposition of fibrinoid acidophilic material drawing serpiginous patterns in the parenchyma (Figure 5). The regular presence of lymphoid follicles around central arterioles. **Pancreas**: interlobular oedema and necrosis of adipocytes, both intralobular and interlobular. No changes were present in the secretory cells. **Stomach**: no significant changes were observed. **Small intestine**: Necrotic enteritis, particularly in the duodenum, with loss of villi and deposition of fibrin in the proximal mucosa (Figure 6). **Kidneys**: the areas of surface retraction in both kidneys corresponded to segmental fibrosis affecting cortex and medulla. In these areas, there was a loss of tubules and glomeruli, which were moderately congested. No microbial agents were identified in any organ and the results of the microbiological analysis were also negative.

### 4.2. Virology

Investigations for MYXV and RHDV (genotypes GI.1b (former G1) to GI.1a (former RHDVa or G6)) [19] were negative. However, the RT-qPCR specific for RHDV2 revealed a high viral load in the liver (approximately 9.0 × 10^11^ copies of RHDV2 RNA per mg of liver), higher than the usual viral load found in infected rabbit liver, including both vaccinated and unvaccinated rabbits [30]. The amplification of the RdRp partial gene and VP60 complete gene was successful, generating amplicons within the expected sizes (available in access number MT829254).

### 4.3. Phylogenetic Analysis

BLAST analysis of VP60 nucleotide sequence showed 96.88% similarity with RHDV2 sequences characterised earlier in 2013 from mainland Portugal, namely with three strains (KF44962, KF44963 and KF44964) collected from wild rabbits originating from the south region, Alentejo and Algarve. BLAST analysis of the partial RdRp gene showed 96.56% similarity with a recombinant RHDV2/NP1 strain (MG763952) collected from a wild rabbit from the north of mainland Portugal in 2015.

BLAST analysis of the 2176 nt long sequence revealed higher similarity with sequence KF442964, obtained from a wild rabbit from South of mainland Portugal sampled in 2013.

**Figure 7:**
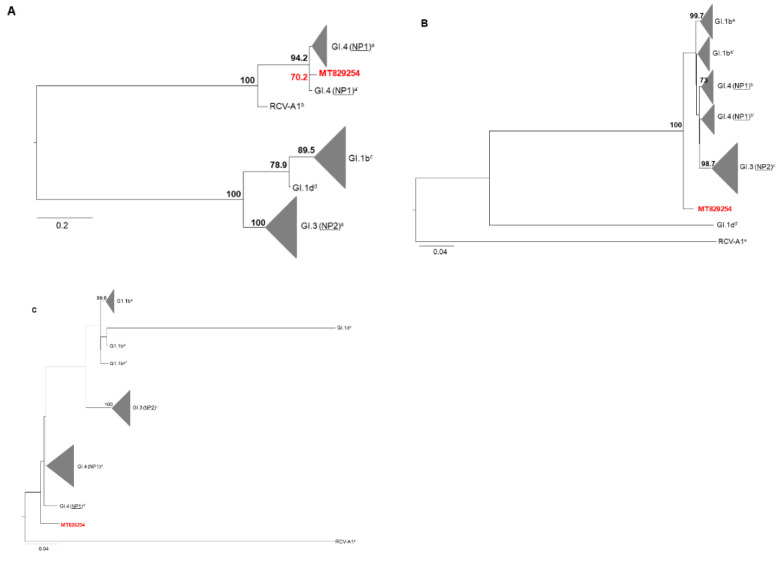
Phylogenetic analysis. (**A**) Maximum likelihood analysis using 14 RdRp gene partial (436 nt) nucleotide sequences, namely GI.4 (NP1–non-pathogenic caliciviruses similar to RCV-A1 are the most likely donors of non-structural proteins) representatives (a-MG763946, MG763944, MG763954 and a’-MG763952), GI.1b (G1) representatives (c-MG763939, MG763938, MG763947 and MG763953), GI.3 (NP2-non-pathogenic caliciviruses similar to CBAnd1 are the most likely donors of non-structural proteins) representatives (e-MG763942, MG763949, MG763943 and MG763945; GI.1d (G3-G5) representatives (d-MH190418) and non-pathogenic rabbit calicivirus Australia 1 (RCV-A1) representatives (b-EU871528). (**B**) Maximum likelihood analysis using 14 complete VP60 gene nucleotide sequences including, GI.1b representatives (a-MG763939 and MG763938 and a’-MG763953 and MG763947), GI.4 (NP1) representatives (b-MG763952 and MG763954 and b’-MG763946 and MG763944), GI.3 (NP2) representatives (c-MG763949, MG763942, MG763945 and MG763943; GI.1d representatives (d-MH190418) and RCVA-A1 representatives (e-EU871528). (**C**) Maximum likelihood analysis using 14 sequences comprising the terminus of the RdRp gene and the complete VP60 gene, namely GI.1b representatives (a-MG763938 and MG763939, a’-MG763947, and a’’-MG763953), GI.3 (NP2) representatives (c-MG763949, MG763942, MG763945 and MG763943), GI.4 (NP1) representatives (d-MG763954, MG763946, MG763944 and d’-MG763952) GI.1d representatives (b-MH190418) and RCVA1 representatives (e-EU871528). The nomenclature used is in accordance with Silvério et al., 2018 [8], Abrantes et al., 2020 [31]. Designations only used by Silvério et al., 2018 are underlined in the phylogenetic trees.

The tree based on the 3′ end sequence of RpRd gene (fragment 436 bp), confirmed that the RHDV2 strain from the dwarf rabbit shared high similarity with the homologous region of the RdRp from other RHDV2 recombinants classified as GI.4 (NP1)/RHDV2 (Figure 7A). The VP60 gene base tree and phylogenetic analysis including the complete fragment showed that the dwarf sequence RHDV2 is in an isolated branch and does not group within the defined clusters (Figure 7B) and (Figure 7C).

Robustness of the tree nodes was assessed by bootstrapping 1000 times. Only bootstrap (BS) values greater or equal to 70 are shown. The graphical representation and edition of the phylogenetic trees were performed with FigTree v1.3.1 (http://tree.bio.ed.ac.uk/software/figtree/).

## 5. Discussion

Rabbit haemorrhagic disease affects both domestic and wild rabbits causing a systemic disease usually with a lethal outcome. After its emergence, RHDV2 strains evolved quickly, with some variation of amino acids in the capsid protein but maintaining the original RHDV2 antigenic profile. Despite this, several RHDV2 recombinants containing the non-structural protein genes of other rabbit lagoviruses (such as GI.1b (G1) and GI.4 or GI.3 (NP-CV)) have been identified in Europe [8]. These include the structural protein (VP60 and VP10) encoding genes of RHDV2 combined with the non-structural protein-encoding genes of GI.1b (RHDV genogroup G1 strain), non-pathogenic rabbit caliciviruses Australia 1 (RCV-A1)-like viruses (GI.4) or other non-pathogenic lagoviruses (GI.3) [8,32,33].

Here we describe an RHDV2 infection in a 2-year-old dwarf rabbit that could have developed an atypical form of RHD before the eventual fatality, four months after the presentation of first signs compatible with the disease. The conclusive diagnosis of RHDV2 was only made post-mortem, making it impossible to claim that the clinical picture observed between February and June was due to RHDV2 infection. However, the clinical signs and the results of the diagnostic investigations (ultrasound hepatic and peri-hepatic changes, an elevated marker of liver injury-ALT, raised total bilirubin and jaundice, hyperglobulinemia and poor coagulation (namely severe subcutaneous haemorrhage and increased prothrombin time (PT) and activated partial thromboplastin time (aPTT)) are all compatible with Rabbit Haemorrhagic Disease. Moreover, no other possible cause for the clinical signs was found. Most of the previous clinical signs, along with a normal hemogram, a leukogram revealing leukopenia and thrombocytopenia have been previously associated with rabbit haemorrhagic disease in a review by Bonvehí et al., 2019 [34].

The atypical development of the clinical course, much longer than the usual, could have resulted from deficient cross-immunity conferred by RHDV vaccination, leading to a subacute disease.

Despite RHDV vaccines do not confer full protection against RHDV2, rabbits vaccinated with RDHV cannot be assumed as immunologically naive against RHDV2 as non-vaccinated animals. The cross-immunity between RHDV and RHDV2 is reported as deficient but not as inexistent [9]. Therefore, it is expected that RHDV-vaccinated animals do not show a primary immune response to RHDV2 infection, since cross-reactive responses (RHDV-RHDV2) to particular epitopes may be beneficial to the protective response, even if deficient.

In fact, the studies on RHDV/RHDV2 cross-immunity are limited to a few strains that emerged soon after 2010. For this reason, the current circulating RHDV2 strains may have different pathogenic characteristics that may explain an unusual presentation in RHDV vaccinated in rabbits. The vaccine used contains a recombinant myxoma virus that expresses the VP60 protein of RHDV, thus can never be responsible for inducing RHD in rabbits. Also, the strain isolated in this animal (RHDV2) is completely different from the one contained in the vaccine (RHDV).

The origin of this infection is unknown. However, in Oeiras district, there are a few populations of wild rabbit that may have been a source of infection for this animal through arthropod vectors. Infection in a hospital context is not probable, considering that there was no case of RHD in that hospital in the last year and that strict disinfection guidelines for spaces and equipment are followed.

The virus identified in this rabbit was confirmed to be a recombinant strain, with the structural backbone of RHDV2 and the non-structural genes of non-pathogenic RCV-A1 strains (GI.4). The clinical course was suggestive, but non-pathognomonic, of rabbit haemorrhagic disease. Post-mortem lesions identified in the liver, spleen and intestine are compatible with an acute clinical course of the disease with fatal outcome. Kidney lesions, on the contrary, are typical of a chronic process, but their severity was not enough to consider renal insufficiency as the cause for the clinical signs.

No studies are evaluating a longer clinical course of the disease caused by RHDV2 recombinant strains such as the one detected in this rabbit. Further studies are necessary to better understand the possibility of different genotypes generating different clinical courses of disease. This case is an important warning to all veterinarians drawing attention to the fact that RHD is an important differential diagnosis to be considered in some clinical settings, and that it is critical to revise and update the vaccination programs towards RHDV2 infection.

## Figures and Tables

**Table 1:** Summary of the rabbit haematological and biochemical parameters and respective reference values retrieved from Carpenter’s 4th ed of the Exotic animal formulary [23] and Idexx’s reference values of the ProCyte Dx* Haematology Analyzer (as of IDEXX VetLab* Station software version 4.48) and Catalyst one Biochemistry analyser, the equipment used in this study.

Haematology	February	March	June	Reference Values	
				Carpenter, 2018	Idexx Lab., 2017
Haematocrit (%)	30.9	36.3	37.6	30–50	29.4–40.9
Haemoglobin (g/dL)	10.7	11.8	13.3	8–17.5	9.8–13.2
Erythrocytes (×10^6^/μL)	5.21	5.67	6.45	4–8	4.45–6.71
MCV (fL)	59.3	64.0	58.3	58–75	58.1–69.6
MCH (pg)	20.5	20.8	20.6	17.5–23.5	18.9–22.1
MCHC (g/dL)	34.6	32.5	35.4	29–37	31.6–33.6
Reticulocytes (%)	2.4	**5.4**	1.4	2–4	-
Reticulocytes (K/μL)	122.4	**306.7** **↑**	88.4	-	69.5–242.7
Platelets (10^3^/μL)	**40** **↓**	370	**97** **↓**	290–650	219–521
WBC (10^3^/μL)	**2.53** **↓**	5.15	**1.86** **↓**	5–12	4.54–10.22
Heterophils (%)	**28.1** **↓**	**28.8** **↓**	47.9	35–55	-
Lymphocytes (%)	**64.4** **↑**	60.0	41.9	25–60	-
Monocytes (%)	4.7	5.4	4.8	2–10	-
Eosinophils (%)	0.8	0.8	0.0	0–5	-
Basophils (%)	2.0	5.0	5.4	2–8	-
Heterophils (K/μL)	**0.71** **↓**	1.48	**0.89** **↓**	-	0.96–3.34
Lymphocytes (K/μL)	1.63	3.09	**0.78** **↓**	-	1.49–5.21
Monocytes (K/μL)	**0.12** **↓**	**0.28** **↓**	**0.09** **↓**	-	0.31–0.99
Eosinophils (K/μL)	**0.02** **↓**	**0.04** **↓**	**0.00** **↓**	-	0.05–2.12
Basophils (K/μL)	**0.05** **↓**	**0.26** **↓**	**0.10** **↓**	-	0.56–2.12
**Biochemistry**					
Glucose	**194** **↑**	-	**164** **↑**	75–150	75–145
Creatinine (CREA)	1.0	-	1.2	0.5–2.6	0.8–1.8
BUN	12	-	22	15–50	10–24
BUN/CREA	12	-	18	-	-
PHOS	-	-	3.7	2.3–6.9	1.2–4.9
CA	-	-	11.7	8–14.8	5.6–12.0
TP	6.6	-	**7.7** **↑**	5.4–7.5	5.5–7.2
ALB	4.4	-	4.4	2.5–5	2.7–4.6
GLOB	2.2	-	**3.3** **↑**	1.5–3.5	1.5–2.8
ALB/GLOB	2.0	-	1.3	-	-
ALT	**240** **↑**	-	**586** **↑**	14–80	31–53
ALKP	99	-	104	4–70	70–145
GGT	-	-	2	-	-
TBIL	-	-	0.9	0–0.75	0.3–0.8
CHOL	-	-	30	12–16	35–53

The values highlighted in bold represent the main deviations observed and the arrows indicate if the values are above (↑) or below (↓) the references.

## Data Availability

The authors confirm that the data supporting the findings of this study are available within the article [and/or] its supplementary materials.

## Supplementary Materials

The following are available online at https://www.mdpi.com/2076-2615/11/1/40/s1, Table S1: Oligonucleotides and protocols used in this study for amplification and sequencing.
