# Supplementary material for: A Potential Atypical Case of Rabbit Haemorrhagic Disease in a Dwarf Rabbit
Source: Animals (Basel). 2020 Dec 28;11(1):40. doi: 10.3390/ani11010040 (PMC7823764; doi:10.3390/ani11010040)
Supplement: Supplementary file 1 [file animals-11-00040-s001.pdf]

Brief Report

A Potential Atypical Case of Rabbit Haemorrhagic Disease in a Dwarf Rabbit

Fábio A. Abade dos Santos [1,2,3,*], Carolina Magro [4], Carina L. Carvalho [2], Pedro Ruivo [5], Margarida D. Duarte [1,2] and Maria C. Peleteiro [1]

[1] Centre for Interdisciplinary Research in Animal Health (CIISA), Faculdade de Medicina Veterinária, Universidade de Lisboa, Avenida da Universidade Técnica, 1300-477 Lisboa, Portugal; mcpelet@fmv.ulisboa.pt (M.C.P.)

[2] Instituto Nacional de Investigação Agrária e Veterinária (INIAV, I.P.), Av. da República, Quinta do Marquês, 2780-157 Oeiras, Portugal; fabio.abade@iniav.pt (F.A.A.S), carina.carvalho@iniav.pt (C.L.C), margarida.duarte@iniav.pt (M.D.D.)

[3] Instituto Universitario de Biotecnología de Asturias (IUBA), Departamento de Bioquímica y Biología Molecular, Universidad de Oviedo, 33006 Oviedo , Spain;

[4] VetOeiras. Hospital Médico-Veterinário, Estrada de Oeiras n18-20, 2780-114 Oeiras, Portugal; carolinamagrovet@gmail.com (C.M.)

[5] Instituto de Medicina Molecular João Lobo Antunes (IMM), Faculdade de Medicina, Universidade de Lisboa, Lisbon, Portugal; ruivo_pedro@hotmail.com (P.R.)

* Correspondence: fabio.abade@iniav.pt

Table S1 –Oligonucleotides and protocols used in this study for amplification and sequencing

Primer name	Primer sequence (5′ to 3′)	Location (using sequence MG763954 as reference)	Size of the amplicon (bp)
UniF	GCCATGACmCCvATGATGGT	4819nt-5472nt	653
5472	CTTGTTGGTCCACTTGTT		
27F	CCATGCCAGACTTGCGTCCC	5792-6290nt	1471
986R	AACCATCTGGAGCAATTTGGG		
717F	CGCAGATCTCCTCACAACCC	6003nt-7071nt	1068
10R	GCGCCTGCAAGTCCCAATCC		

Amplification of the full vp60 gene was carried out using two pairs of primers, 27F (5′-CTCGGTAGTACCTGACGACG-3′) 3 and 986R (5′-AACCATCTGGAGCAATTTGGG-3′) 2, and 717F (5′-CGCAGATCTCCTCACAACCC-3′ 2 and RC10R 4. Two overlapping fragments were obtained. The PCR reactions were performed with 2µl (tenho de confirmar) of cDNA and 0.5µM of each primer, using the Phusion™ High–Fidelity DNA Polymerase kit (ThermoFisher Scientific), according to the manufacturer's protocol. Amplification conditions included an initial denaturation at 98°C for 35 seconds, followed by 35 cycles of denaturation at 98°C for 10 seconds, annealing at 60°C for 30 seconds and extension at 72°C for 30 seconds. The final extension step was carried out at 72ºC for 7 minutes. The fragments were excised from agarose gel after electrophoresis and purified with the NZYGelpure (Nzytech genes and enzymes, Lisbon, Portugal). Sequencing of the vp60 gene was performed with the amplifying primers described above using the BigDyeTM Terminator cycle sequencing kit (Applied Bosystems, Foster City, CA, USA), according with the manufacturer's instructions.

To further investigate if the strain under characterization corresponded to a pure RHDV2 strain (RHDV2 non-structural and RHDV2 structural genes) or to a RHDV2 recombinant, a genomic region located upstream the vp60 gene, including the 3′ end of the rdrp gene (a non-structural gene) was amplified by conventional PCR. Amplification was carried out using the pair of primers Uni-F 1 and 5274R (This study), designed by our team, to obtain a xxxbp fragment. The PCR reactions were performed with 2µl (confirmar) of cDNA and 0.5µM of each primer, using the Phusion™ High–Fidelity DNA Polymerase (ThermoFisher Scientific), according to the manufacturer's protocol. Amplification conditions included an initial denaturation at 98°C for 35 seconds, followed by 35 cycles of denaturation at 98°C for 10 seconds, annealing at 55°C for 30 seconds and extension at 72°C for 30 seconds. The final extension step was carried out at 72ºC for 7 minutes. The fragment was excised from agarose gel after electrophoresis and purified with the NZYGelpure (Nzytech genes and enzymes, Lisbon, Portugal).

References

1. Dalton KP, Arnal JL, Benito AA, Chacón G, Martín Alonso JM, Parra F. Conventional and real time RT-PCR assays for the detection and differentiation of variant rabbit hemorrhagic disease virus (RHDVb) and its recombinants. *J Virol Methods*. 2018;251:118–122.
2. Duarte M, Carvalho C, Bernardo S, et al. Rabbit haemorrhagic disease virus 2 (RHDV2) outbreak in Azores: Disclosure of common genetic markers and phylogenetic segregation within the European strains. *Infect Genet Evol*. 2015;35:163–171.
3. Le Gall G, Arnauld C, Boilletot E, Morisse JP, Rasschaert D. Molecular epidemiology of rabbit haemorrhagic disease virus outbreaks in France during 1988 to 1995. *J Gen Virol*. 1998;79:11–16.
4. Tham KM, Barnes SM, Hunter SN. Polymerase chain reaction amplification and gene sequence analysis of a calicivirus from a feral rabbit. *Virus Genes*. 1999;18:235–242.
